# Trace element imbalance and oxidative-inflammatory dysregulation in women with endometriosis

**DOI:** 10.3389/fendo.2026.1973603

**Published:** 2026-10-29

**Authors:** Ashish Ashish, Sangeeta Rai, Shivani Mishra, Anil Kumar Maurya, Shani Vishwakarma, Nitish Kumar Singh, Abhay Kumar Yadav, Royana Singh

**Affiliations:** 1 Multidisciplinary Research Unit, ICMR-DHR, Institute of Medical Sciences, Banaras Hindu University, Varanasi, India; 2 Department of Obstetrics & Gynaecology, Institute of Medical Sciences, Banaras Hindu University, Varanasi, India; 3 Department of Anatomy, Institute of Medical Sciences, Banaras Hindu University, Varanasi, India

**Keywords:** cytokines, endometriosis, IL-1β, IL-6, immune dysregulation, oxidative stress, redox homeostasis, TNF-α

## Abstract

**Background:**

Endometriosis is a chronic inflammatory and estrogen-dependent disorder associated with oxidative stress, immune dysregulation, and altered trace-element homeostasis. This study investigated the association between serum trace elements, oxidative-stress biomarkers, and inflammatory cytokines in women with endometriosis, with particular emphasis on their interplay in disease pathophysiology and their potential utility for integrated biomarker profiling.

**Materials and Methods:**

A total of 120 women, comprising 60 women with endometriosis and 60 healthy controls, were enrolled. Serum concentrations of trace elements (As, Pb, Cd, Hg, Cu, Fe, Zn, and Se) were measured using graphite furnace atomic absorption spectrophotometry. Oxidative-stress biomarkers (MDA, SOD, glutathione, and 8-OHdG) and inflammatory cytokines (TNF-α, TGF-β, IFN-γ, IL-6, IL-1β, and IL-10) were assessed using ELISA-based methods. Data distribution was assessed using the Shapiro–Wilk test before group comparisons. Trace-element and oxidative-stress biomarkers showed non-normal distributions and were analyzed using the Mann–Whitney U test, whereas inflammatory cytokine variables met normality assumptions and were analyzed using the unpaired Student’s t-test. Associations among biomarkers were evaluated using Spearman’s rank correlation analysis.

**Results:**

Women with endometriosis exhibited significantly higher serum levels of As, Pb, Hg, Cu, and Fe and significantly lower Zn and Se levels compared with healthy controls. Oxidative-stress profiling demonstrated significantly increased MDA, SOD, and 8-OHdG levels and significantly reduced glutathione levels in the endometriosis group (*p* < 0.001). Inflammatory profiling revealed significantly elevated TNF-α, IL-6, IL-1β, and IL-10 levels, whereas IFN-γ was significantly reduced; TGF-β showed a non-significant increasing trend. Spearman’s correlation analysis identified selective associations among trace elements, oxidative-stress biomarkers, and inflammatory cytokines, including positive correlations of Cu with IL-1β and As with TGF-β, and a negative correlation of Fe with glutathione.

**Conclusion:**

Women with endometriosis demonstrated coordinated alterations in trace-element status, redox homeostasis, and inflammatory signaling. The observed associations suggest an interconnected redox-inflammatory phenotype that may contribute to the biological stress environment associated with endometriosis. These findings support further investigation of trace-element and oxidative-stress profiles as potential biomarkers of disease-related biological alterations, while larger multicenter and longitudinal studies are needed to establish their clinical relevance and causal relationships.

## Graphical Abstract

**Figure:**
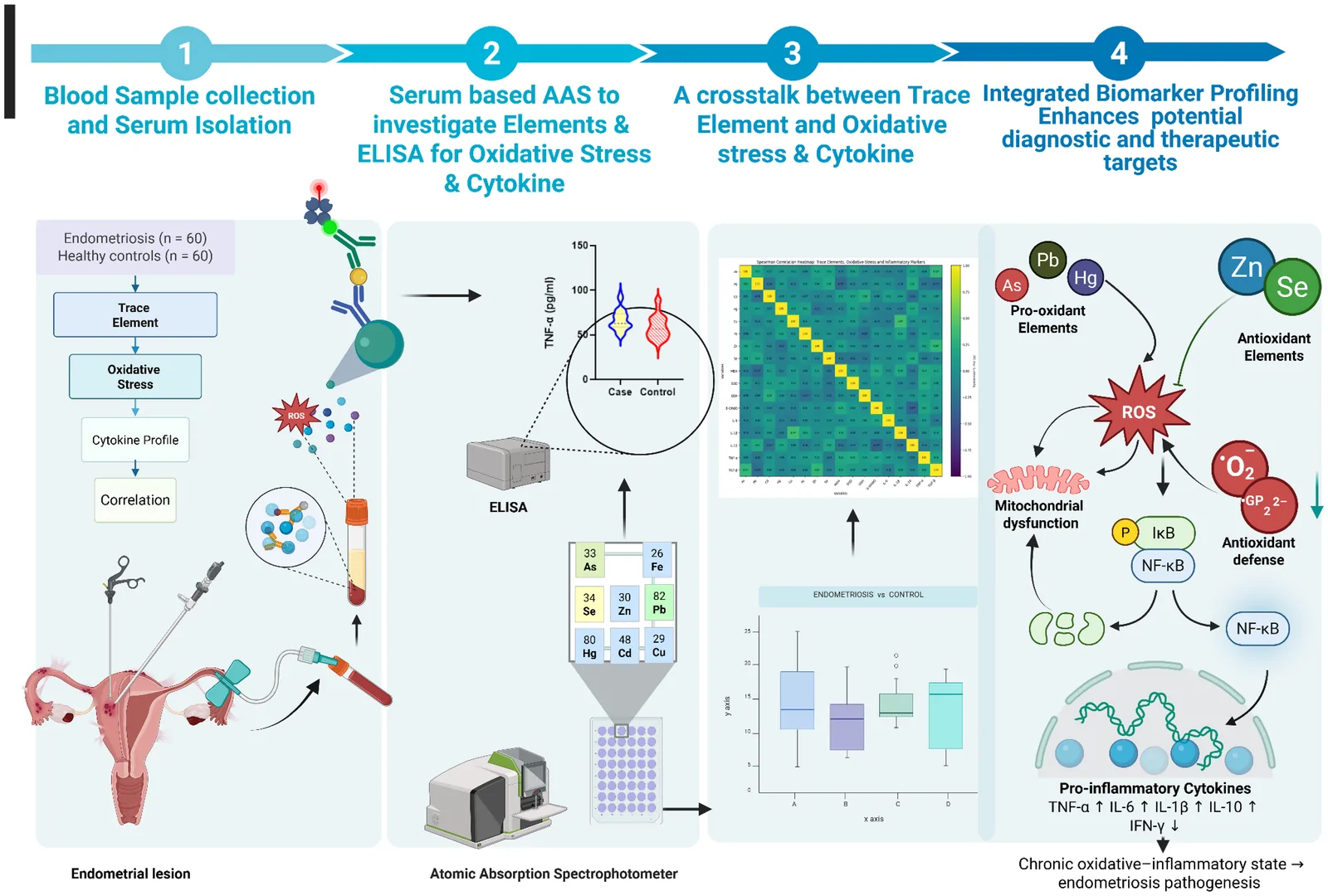

## Introduction

Endometriosis is a chronic inflammatory condition characterized by persistent immune activation, oxidative stress, and altered tissue homeostasis (1). These processes may influence cellular homeostasis, defined as the capacity to maintain or restore physiological homeostasis in response to cellular stress. Essential trace elements such as zinc and selenium contribute to antioxidant defense, whereas exposure to potentially toxic elements such as lead, mercury, and arsenic may increase oxidative and inflammatory burden (2). The convergence of chronic inflammation, oxidative stress, and altered trace-element homeostasis highlights the complex molecular interplay underlying endometriosis and provides a strong rationale for investigating their interconnected roles in disease pathophysiology and integrated biomarker profiling (3). Therefore, the present study investigated circulating trace elements together with oxidative-stress biomarkers and inflammatory cytokines in women with endometriosis.

### Trace-element homeostasis, oxidative stress, and inflammatory signalling in endometriosis

Persistent immune activation, oxidative stress, and disruption of tissue homeostasis are central features of endometriosis and contribute to its complex molecular pathology (4). Essential trace elements such as zinc and selenium play important roles in antioxidant defense and maintenance of redox balance, whereas potentially toxic elements including lead, mercury, and arsenic may increase oxidative and inflammatory burden when their concentrations are altered (5–7). These disturbances may amplify oxidative damage, impair antioxidant defenses, and influence inflammatory signaling pathways involved in lesion establishment and disease progression (8, 9). Accordingly, investigating the interplay between trace-element homeostasis, oxidative-stress biomarkers, and inflammatory cytokines may provide mechanistic insights into endometriosis pathophysiology and support the development of integrated biomarker profiles for improved disease characterization (9).

## Methodology

This case–control study was conducted from January 2024 to January 2025 at the Maternal and Child Health (MCH) Centre, Department of Obstetrics and Gynaecology, Sir Sunder Lal Hospital, Institute of Medical Sciences, Banaras Hindu University. Ethical approval for the study was obtained from the Institutional Ethical Committee of the Institute of Medical Sciences, Banaras Hindu University (Approval No. Dean/2021/EC/9397). Written informed consent was obtained from all participants, including both endometriosis patients and healthy controls, prior to sample collection. Endometriosis staging was performed according to the revised American Society for Reproductive Medicine (ASRM) classification, and participants with stage I–IV disease were included (10, 11). The distribution of disease severity among patients was as follows: Stage I (22.5%), Stage II (27.5%), Stage III (30.0%), and Stage IV (20.0%). Thus, 50% of cases were categorized as moderate-to-severe disease (Stage III–IV). Clinically, ovarian endometrioma was present in 48% of patients, peritoneal lesions in 32%, and deep infiltrating endometriosis (DIE) in 20% of cases.

A total of 135 women were initially screened, of whom 120 participants (60 women with endometriosis and 60 healthy controls) were included in the final analysis after application of the eligibility criteria. Healthy controls were fertile women without clinical or laparoscopic evidence of endometriosis, with regular menstrual cycles and no history of infertility or pelvic pain. We selected fertile women without clinical or laparoscopic evidence of endometriosis as controls to minimize the likelihood of undiagnosed disease. However, because infertility was part of the case definition whereas controls were fertile, this design may introduce selection bias and should be considered when interpreting between-group comparisons. Sample size was calculated using G*Power version 3.1, assuming a moderate effect size (Cohen’s d = 0.60), α = 0.05, and 80% power, yielding a minimum requirement of 45 participants per group. To account for exclusions and improve statistical robustness, 60 participants were included in each group. Blood samples were collected during routine clinical evaluation. Menstrual-cycle phase was not standardized, and this is acknowledged as a limitation because cyclic hormonal variation may influence inflammatory and oxidative-stress biomarkers. Serum samples were stored at −80 °C until analysis.

### Inclusion exclusion criteria

Inclusion: Women aged 18–46 with laparoscopic/histopathological diagnosis of endometriosis; no hormonal/anti-inflammatory treatment in past 3 months; Infertility due to endometriosis. Exclusion: Obesity (BMI > 30), known metabolic disorders, lipid abnormalities, pregnancy or lactation, recent surgery, chronic or acute infection, immunosuppressant or antioxidant use, history of cancer, diabetes, cardiovascular disorders, smoking, alcohol abuse, or incomplete clinical data.

### Preparation of serum/diluent/standard and blank

Serum samples were analyzed using graphite furnace atomic absorption spectrophotometry (GFAAS) without complete mineralization. This approach was adopted to minimize analyte loss, reduce contamination risk, and avoid prolonged digestion procedures, as direct dilution is a validated method for serum analysis using GFAAS. Previous studies have demonstrated that direct dilution coupled with graphite furnace AAS provides reliable quantification of trace elements in serum while minimizing analyte loss and contamination associated with acid digestion procedures. Chavan et al. (2022) (12) successfully applied a similar strategy for serum trace element estimation using GFAAS. In the present study, analytical reliability was ensured through calibration with certified standard reference solutions and repeated quality-control measurements. Owing to the high sensitivity of GFAAS and its suitability for small biological sample volumes, a direct dilution method was employed to minimize matrix interference while preserving analyte stability. A diluent was prepared by mixing 50 µL of 1% (v/v) trace-metal grade nitric acid and 200 µL of 0.4% Triton X in 49.75 mL of ultrapure deionized water (18.2 MΩ·cm) 50 mL Falcon tube, followed by thorough mixing. All reagents were of analytical grade; trace-metal grade nitric acid (≥69%, Merck) was used. For standard preparation, 50 µL of stock solution was diluted in 49.95 mL of 1% nitric acid to obtain a 1 ppm standard, while 0.2 mL of stock was diluted in 9.8 mL of 1% nitric acid for a 20-ppb standard. A blank was prepared by adding 200 µL of 1% nitric acid to 800 µL of the diluent in a sampler tube. These steps ensure accurate calibration and baseline readings for AAS analysis.

### Analysis of serum trace element

Serum levels of trace elements As, Pb, Cd, Hg, Cu, Fe, Zn, and Se—were measured using the Perkin Elmer Pinnacle 900T Atomic Absorption Spectrophotometer (Graphite Furnace method) in the Department of Anatomy, IMS, BHU, following the procedure by Chavan S,et al. (2022). Graphite furnace atomic absorption spectrophotometry (GFAAS) was performed using element-specific hollow cathode lamps under optimized instrumental conditions. The analytical wavelengths (nm) used were: As (193.7), Pb (283.3), Cd (228.8), Hg (253.7), Cu (324.8), Fe (248.3), Zn (213.9), and Se (196.0). Background correction was applied using the Zeeman correction system. The graphite furnace temperature program included sequential drying (110–130 °C), ashing (400–900 °C), and atomization (1800–2400 °C) stages as per manufacturer recommendations. Calibration curves were prepared using certified standard solutions with correlation coefficients (R²) > 0.995. Method validation was performed to ensure analytical reliability. Limits of detection (LOD) and limits of quantification (LOQ) were calculated as 3σ/s and 10σ/s, respectively, where σ represents the standard deviation of the blank and s is the slope of the calibration curve. Intra-assay precision was evaluated by analysing samples in triplicate, and the coefficient of variation (CV%) was maintained below 5%. Calibration accuracy was verified using certified standard reference solutions. Commercially available certified reference materials (CRMs) and certified standard solutions supplied by PerkinElmer/Merck were used for instrument calibration and quality assurance. Recovery and reproducibility were monitored through triplicate analyses and periodic blank measurements. Analytical performance was further ensured through the use of certified reference standards, calibration verification, triplicate sample analysis, and routine quality-control procedures throughout the study.

### Analysis of oxidative stress and antioxidant biomarkers

Serum levels of Oxidative Stress were measured using commercially available enzyme-linked immunosorbent assay (ELISA) kits from ELK Biotechnology Co. LTD. The following specific kits were used for quantification: MDA (Malondialdehyde), SOD (Superoxide Dismutase), Glutathione (GSH), and 8-OHdG (8-Hydroxy-2’-deoxyguanosine). All assays were performed following the manufacturer’s instructions, and absorbance was measured using a micro plate reader. Standard curves were generated for each marker to ensure accurate quantification.

### Analysis of inflammatory cytokines

Serum levels of inflammatory cytokines including TNF-α, TGF-β, IFN-γ, IL-6, IL-1β, and IL-10 were measured using commercially available enzyme-linked immunosorbent assay (ELISA) kits obtained from ELK Biotechnology Co., Ltd. All experimental procedures were carried out at the Multidisciplinary Research Unit (MRU), Institute of Medical Sciences, Banaras Hindu University. Serum samples were processed according to the manufacturer’s instructions, and absorbance was measured using a microplate reader at 450 nm. Cytokine concentrations were calculated using standard calibration curves and expressed as pg/mL. All samples were analyzed in duplicate to ensure analytical accuracy and reproducibility.

### Statistical analysis

Statistical analyses were performed using SPSS software version 26.0 (IBM Corp., Armonk, NY, USA) (13). Data distribution for each variable was assessed using the Shapiro–Wilk test prior to selecting the appropriate statistical test. Trace-element and oxidative-stress biomarkers exhibited non-normal distributions and were therefore compared using the Mann–Whitney U test. In contrast, all inflammatory cytokine variables satisfied the Shapiro–Wilk normality test and were therefore compared using the unpaired Student’s t-test. Effect sizes (r) were calculated for non-parametric comparisons and interpreted according to Cohen’s criteria as small (0.10–0.29), medium (0.30–0.49), and large (≥0.50). Spearman’s rank correlation analysis was performed to evaluate associations among trace elements, oxidative-stress biomarkers, and inflammatory cytokines because several variables did not follow a normal distribution. Because this was an exploratory cross-sectional case-control study, multivariable regression analyses adjusting for age and fertility status were not performed; therefore, these variables are acknowledged as potential sources of residual confounding. A two-tailed p-value of <0.05 was considered statistically significant. Given the exploratory nature of the correlation analysis and the large number of pairwise comparisons, the reported correlation findings were interpreted cautiously, and emphasis was placed on the magnitude and biological plausibility of the associations rather than statistical significance alone. No formal correction for multiple comparisons was applied because the correlation analysis was exploratory; therefore, nominal p-values should be interpreted cautiously and require validation in independent cohorts.

## Results

A total of 135 participants were initially screened, of whom 120 participants (60 women with endometriosis and 60 healthy controls) were included in the final analysis. Clinical and biochemical characteristics are presented in **Table 1**. Compared with healthy controls, women with endometriosis had significantly higher serum levels of LH, FSH, prolactin, T4, TSH, and CA-125 (p < 0.05 for all), whereas T3 and BMI did not differ significantly between groups. The endometriosis group was significantly younger than the control group (29.13 ± 5.44 vs. 32.40 ± 8.44 years, p = 0.013).

**Table 1:** Statistical Comparison of Hormonal and Clinical Parameters Between Endometriosis Cases and Controls.

Variable	Group	Mean (n=60)	Std. deviation	Range	T	p-value
Age (years)	Case	29.13	5.441	18 - 46	2.521	**0.013**
	Control	32.40	8.436	18 - 45		
BMI (kg/m²)	Case	21.52	2.157	17.2 - 25.8	.127	0.899
	Control	21.47	2.350	18.2 - 29.9		
LH (IU/L)	Case	6.79	2.983	2.46 - 15.10	8.462	**<0.001**
	Control	3.06	1.666	0.67 - 7.45		
FSH (IU/L)	Case	12.33	6.869	1.88 - 22.51	6.716	**<0.001**
	Control	6.22	1.602	3.60 - 9.30		
Prolactin (ng/mL)	Case	17.27	4.284	3.10 - 41.90	11.619	**<0.001**
	Control	9.20	3.264	3.80 - 15.20		
T3 (ng/dL)	Case	129.20	25.727	84.0 - 205.0	.091	0.928
	Control	129.72	35.848	67.0 - 198.0		
T4 (μg/dL)	Case	9.50	2.706	4.89 - 20.40	3.918	**<0.001**
	Control	7.77	2.077	4.50 - 12.30		
TSH (μIU/mL)	Case	3.20	3.271	0.39 - 23.32	2.159	**0.033**
	Control	2.24	1.073	0.40 - 4.00		
CA-125 (U/mL)	Case	36.80	8.720	22.5 - 68.0	8.566	**<0.001**
	Control	20.65	11.717	1.0 - 45.0		

Bold values indicate statistically significant differences between the endometriosis and control groups (p < 0.05).

The Mann–Whitney U test revealed significant differences between the case and control groups for most trace elements. Specifically, the case group had significantly higher mean ranks for arsenic (As), U = 1169.50, Z = −3.31, p = .001, r = .30; lead (Pb), U = 620.50, Z = −6.19, p <.001, r = .57; mercury (Hg), U = 970.00, Z = −4.36, p <.001, r = .40; copper (Cu), U = 952.50, Z = −4.45, p <.001, r = .41; and iron (Fe), U = 747.50, Z = −5.52, p <.001, r = .50, compared with the control group. Conversely, the control group exhibited significantly higher mean ranks for zinc (Zn), U = 906.50, Z = −4.69, p <.001, r = .43, and selenium (Se), U = 748.00, Z = −5.52, p <.001, r = .50. However, no statistically significant difference was observed for cadmium (Cd), U = 1458.00, Z = −1.80, p = .073, r = .16. These findings demonstrate significant differences in selected serum trace-element levels between women with endometriosis and controls as shown in **Figure 1**, **Table 2**.

**Figure 1:**
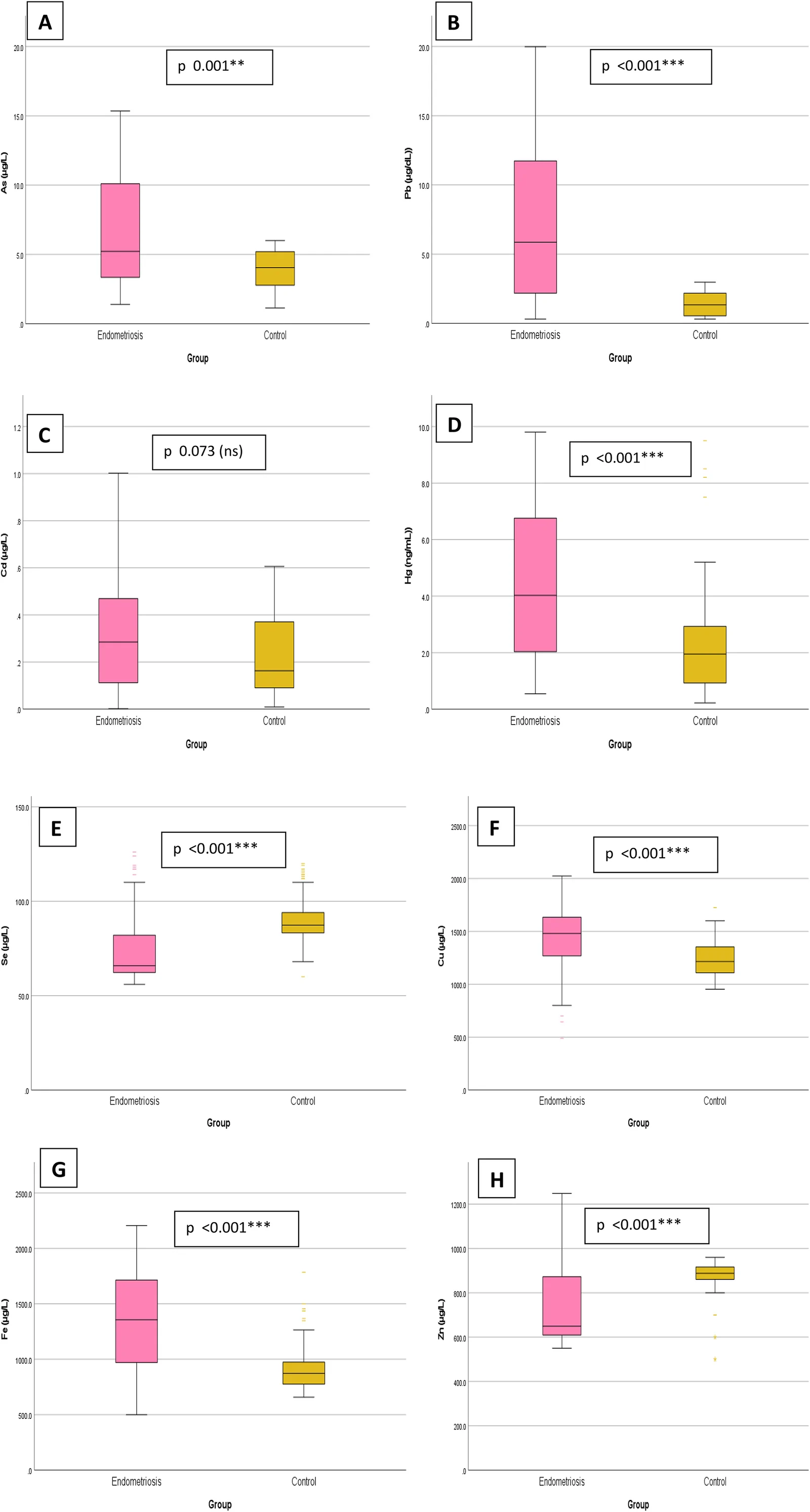
Comparison of serum trace element concentrations between women with endometriosis (n = 60) and healthy controls (n = 60). Data are presented as median (IQR). Statistical significance was assessed using the Mann–Whitney U test (*p < 0.05, **p < 0.01, ***p < 0.001; ns, not significant). Panels represent **(A)** As, **(B)** Pb, **(C)** Cd, **(D)** Hg, **(E)** Se, **(F)** Cu, **(G)** Fe, and **(H)** Zn.

**Table 2:** Mann–Whitney U test comparing trace element levels between cases and controls (N = 120).

Trace element	Case mean rank	Control mean rank	Mann–Whitney U	Z	*P*	Effect size (*r*)	LOD	LOQ
As (µg/L)	71.01	49.99	1169.50	-3.309	p.001	0.30	0.60	2.00
Pb (µg/dL)	80.16	40.84	620.50	-6.194	p < 0.001	0.57	0.30	1.00
Cd (µg/L)	66.20	54.80	1458.00	-1.795	p.073	0.16	0.10	0.33
Hg (ng/mL)	74.33	46.67	970.00	-4.357	p < 0.001	0.40	0.30	1.00
Cu (µg/L)	74.63	46.38	952.50	-4.448	p < 0.001	0.41	3.00	10.00
Fe (µg/L)	78.04	42.96	747.50	-5.524	p < 0.001	0.50	3.00	10.00
Zn (µg/L)	45.61	75.39	906.50	-4.690	p < 0.001	0.43	3.00	10.00
Se (µg/L)	42.97	78.03	748.00	-5.522	p < 0.001	0.50	1.20	4.00

Values represent mean ranks obtained from the Mann–Whitney U test. LOD, limit of detection; LOQ, limit of quantification; r, effect size. Statistical significance was set at p < 0.05.

The Mann–Whitney *U* test revealed statistically significant differences between the case and control groups for all oxidative stress biomarkers examined. The case group demonstrated significantly higher mean ranks for malondialdehyde (MDA), *U* = 627.00, *Z* = −6.16, *p* <.001, *r* = .56; superoxide dismutase (SOD), *U* = 0.00, *Z* = −9.45, *p* <.001, *r* = .86; and 8-hydroxy-2′-deoxyguanosine (8-OHdG), *U* = 540.50, *Z* = −6.61, *p* <.001, *r* = .60, compared with the control group. In contrast, the control group exhibited significantly higher mean ranks for glutathione, *U* = 309.50, *Z* = −7.83, *p* <.001, *r* = .71. These findings demonstrate a distinct oxidative-stress profile in women with endometriosis (**Figure 2****;** **Table 3****).**

**Figure 2:**
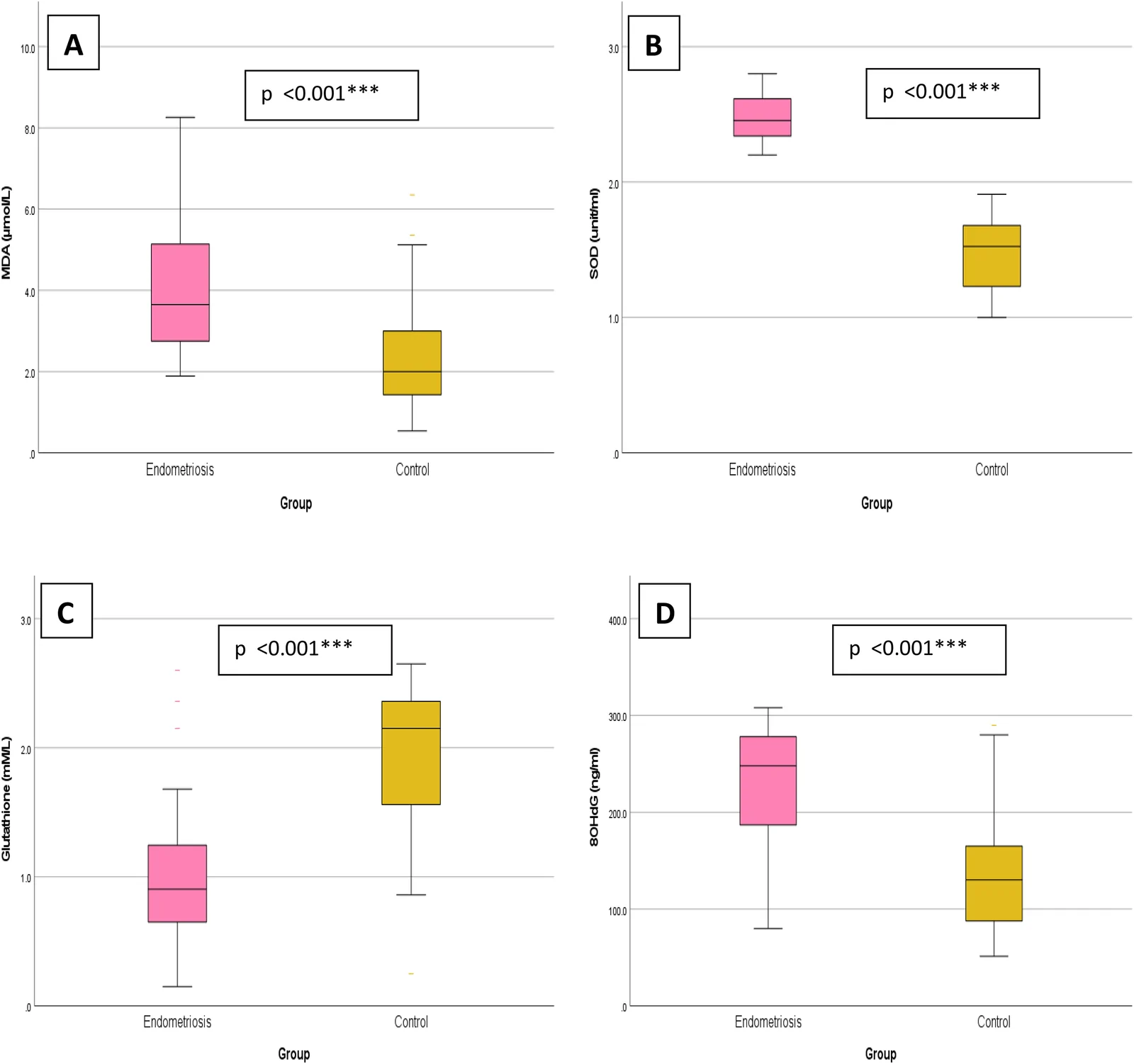
Comparison of oxidative stress biomarkers between women with endometriosis (n = 60) and healthy controls (n = 60). Serum levels of **(A)** MDA, **(B)** SOD, **(C)** glutathione, and **(D)** 8-OHdG were measured by ELISA. Data are presented as median (IQR). Statistical significance was determined using the Mann–Whitney U test (*p < 0.05, **p < 0.01, ***p < 0.001).

**Table 3:** Mann–Whitney U test comparing oxidative stress biomarkers between women with endometriosis and healthy controls (N = 120).

Biomarker	Case mean rank	Control mean rank	Mann–Whitney U	Z	*P*	Effect size (*r*)
MDA (µmol/L)	80.05	40.95	627.00	-6.158	p < 0.001	0.56
SOD (U/mL)	90.50	30.50	0.00	-9.448	p < 0.001	0.86
Glutathione (mM/L)	35.66	85.34	309.50	-7.828	p < 0.001	0.71
8-OHdG (ng/mL)	81.49	39.51	540.50	-6.611	p < 0.001	0.60

Values represent the mean ranks for the case and control groups obtained from the Mann–Whitney *U* test. Two-tailed *p* values are reported, with statistical significance set at *p* <.05.

The levels of oxidative stress biomarkers (MDA, SOD, glutathione, and 8-OHdG) differed significantly between women with endometriosis and healthy controls (all p < 0.001). The endometriosis group showed significantly higher levels of MDA, SOD, and 8-OHdG, with large effect sizes (r = 0.56, 0.86, and 0.60, respectively), whereas glutathione levels were significantly lower in the endometriosis group (r = 0.71). All inflammatory cytokine variables satisfied the Shapiro–Wilk normality assumption; therefore, group comparisons were performed using the unpaired Student’s t-test.

Serum levels of TNF-α, IL-6, IL-1β, and IL-10 were significantly elevated in endometriosis cases compared to controls (p < 0.01). IFN-γ levels were significantly reduced in endometriosis cases, suggesting altered immune regulation and inflammatory imbalance. Although TGF-β levels were comparatively higher in cases, the difference was not statistically significant (p = 0.0736). These findings indicate an altered inflammatory and immune cytokine profile in women with endometriosis (**Figure 3**; **Table 4****).**

**Figure 3:**
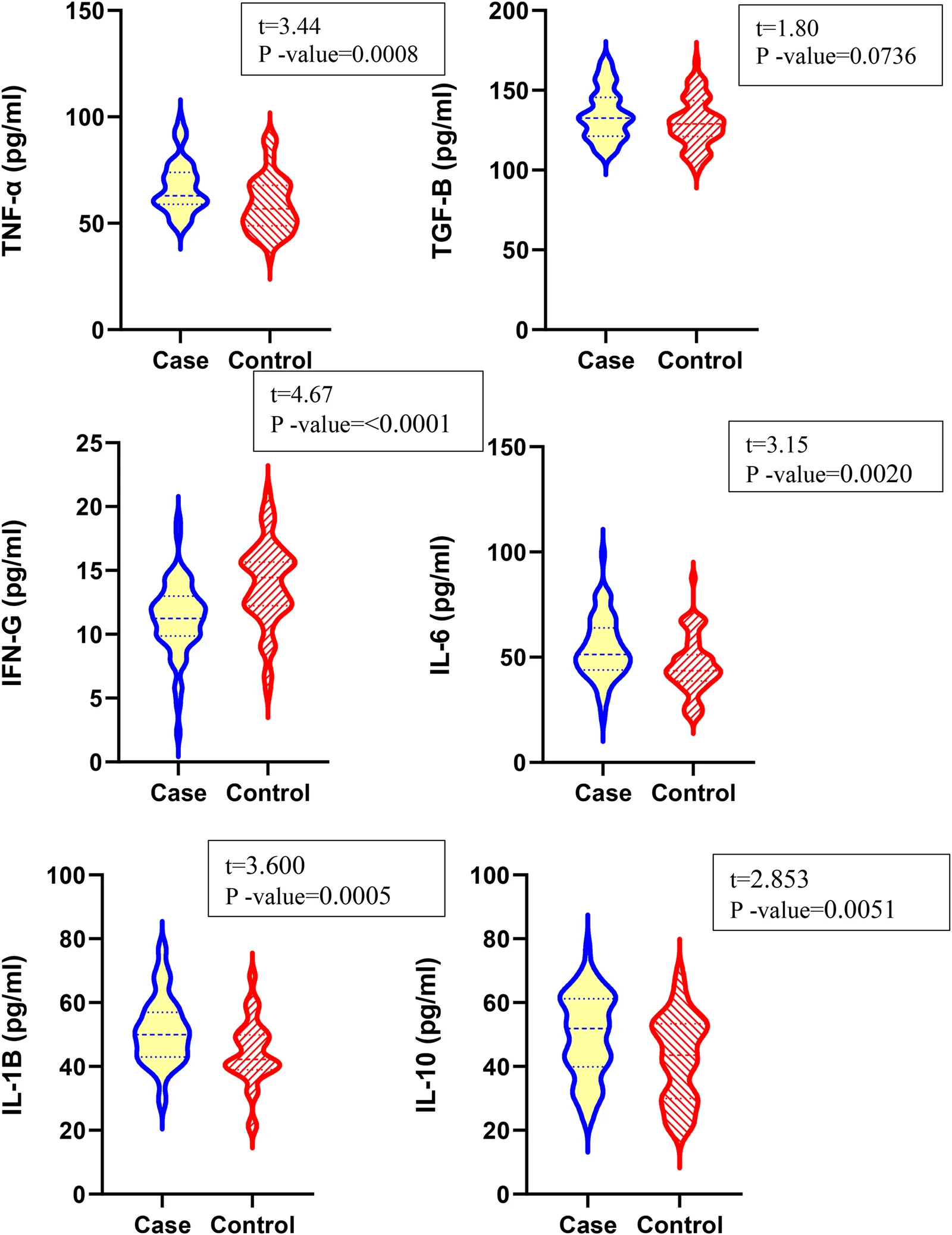
Comparative analysis of inflammatory cytokine levels between endometriosis cases and controls. Serum concentrations of TNF-α, TGF-β, IFN-γ, IL-6, IL-1β, and IL-10 were measured by ELISA. Data are expressed as mean ± SEM. Statistical significance was determined using the unpaired Student’s t-test after confirmation of normality using the Shapiro–Wilk test.

**Table 4:** Comparison of inflammatory cytokine levels between endometriosis cases and controls using the unpaired Student’s t-test.

Inflammatory cytokine (pg/mL)	Case group (mean ± S.E.M)	Control group (mean ± S.E.M)	t-value	df	P-value
TNFα	66.93 ± 2.353	58.82 ± 2.10	3.447	118	0.0008***
TGF-β	134.8 ± 2.847	129.6 ± 3.12	1.805	118	0.0736
IFN-γ	11.27 ± 0.5713	13.95 ± 0.63	4.678	118	<0.0001****
IL-6	54.25 ± 2.600	46.04 ± 2.28	3.158	118	0.0020**
IL-1β	51.24 ± 1.883	44.46 ± 1.74	3.600	118	0.0005***
IL-10	49.65 ± 2.420	42.75 ± 2.05	2.853	118	0.0051**

Data are presented as mean ± SEM. Normality of cytokine variables was confirmed using the Shapiro–Wilk test; therefore, statistical significance was assessed using the unpaired Student’s t-test. Significance levels are indicated as *p < 0.05, **p < 0.01, ***p < 0.001, and ****p < 0.0001.

The Spearman correlation analysis demonstrated several statistically significant associations among trace elements, oxidative stress markers, and inflammatory cytokines in women with endometriosis. Because this was an exploratory cross-sectional case-control study, multivariable regression analyses adjusting for age and fertility status were not performed; therefore, these variables are acknowledged as potential sources of residual confounding. Arsenic (As) showed a significant positive correlation with TGF-β (ρ = .322, p = .012), indicating that higher As concentrations were associated with higher TGF-β levels. Similarly, zinc (Zn) was positively correlated with TGF-β (ρ = .305, p = .018). Lead (Pb) demonstrated a significant negative correlation with MDA (ρ = −.305, p = .018) and with TGF-β (ρ = −.288, p = .026) **Figure 4**, **Table 5**. These findings indicate that higher Pb concentrations were associated with lower levels of MDA and TGF-β in the study sample. Copper (Cu) showed a moderate positive correlation with IL-1β (ρ = .389, p = .002), suggesting an association between Cu concentrations and IL-1β-related inflammatory activity. Iron (Fe) demonstrated a significant negative correlation with glutathione (ρ = −.258, p = .047), indicating that higher Fe levels were associated with lower glutathione concentrations. Among the oxidative stress and inflammatory markers, 8-OHdG was significantly negatively correlated with IL-10 (ρ = −.368, p = .004). In addition, IL-1β showed a significant negative correlation with IL-10 (ρ = −.255, p = .049). These findings suggest an inverse relationship between the oxidative DNA damage marker 8-OHdG and the anti-inflammatory cytokine IL-10.

**Figure 4:**
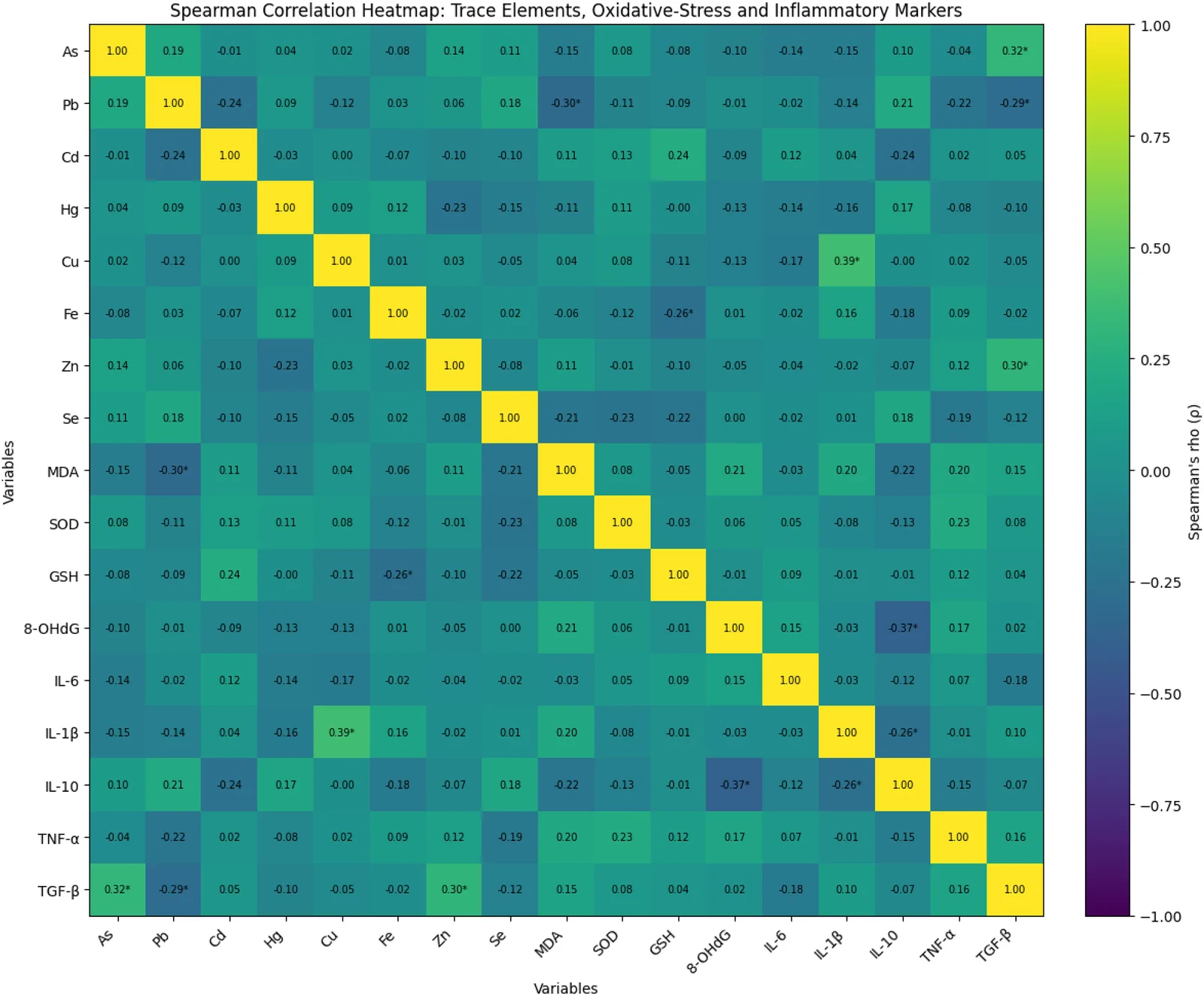
Spearman correlation heatmap showing the relationships among serum trace elements, oxidative-stress biomarkers, and inflammatory cytokines in women with endometriosis (n = 60). Values represent Spearman correlation coefficients (ρ). Positive correlations are shown in green/yellow shades, whereas negative correlations are shown in blue/purple shades, with color intensity representing the strength of the association. *p < 0.05 indicates a statistically significant correlation.

**Table 5:** Spearman’s rank correlation coefficients among trace elements, oxidative-stress biomarkers, and inflammatory cytokines in women with endometriosis (n = 60).

Variable	As	Pb	Cd	Hg	Cu	Fe	Zn	Se	MDA	SOD	Glutathione	8-OHdG	IL-6	IL-1β	IL-10	TNF-α	TGF-β
As (µg/L)	1.000	.188	-.006	.036	.023	-.081	.141	.110	-.147	.081	-.078	-.105	-.141	-.146	.096	-.037	.322*
Pb (µg/dL)	.188	1.000	-.244	.085	-.124	.029	.058	.181	-.305*	-.108	-.085	-.014	-.019	-.138	.212	-.222	-.288*
Cd (µg/L)	-.006	-.244	1.000	-.025	.001	-.072	-.102	-.099	.106	.127	.240	-.088	.117	.045	-.244	.024	.052
Hg (ng/mL)	.036	.085	-.025	1.000	.086	.124	-.235	-.148	-.107	.106	-.002	-.132	-.144	-.164	.171	-.079	-.105
Cu (µg/L)	.023	-.124	.001	.086	1.000	.005	.025	-.047	.038	.077	-.106	-.129	-.174	**.389**	-.004	.023	-.054
Fe (µg/L)	-.081	.029	-.072	.124	.005	1.000	-.017	.018	-.064	-.115	-.258*	.005	-.023	.162	-.185	.092	-.020
Zn (µg/L)	.141	.058	-.102	-.235	.025	-.017	1.000	-.076	.110	-.009	-.096	-.054	-.036	-.019	-.068	.118	.305*
Se (µg/L)	.110	.181	-.099	-.148	-.047	.018	-.076	1.000	-.211	-.228	-.218	.002	-.018	.008	.182	-.192	-.119
MDA (µmol/L)	-.147	-.305*	.106	-.107	.038	-.064	.110	-.211	1.000	.080	-.052	.209	-.025	.201	-.220	.199	.151
SOD (unit/ml)	.081	-.108	.127	.106	.077	-.115	-.009	-.228	.080	1.000	-.026	.057	.047	-.076	-.129	.225	.083
Glutathione (mM/L)	-.078	-.085	.240	-.002	-.106	-.258*	-.096	-.218	-.052	-.026	1.000	-.012	.090	-.011	-.011	.115	.040
8-OHdG (ng/ml)	-.105	-.014	-.088	-.132	-.129	.005	-.054	.002	.209	.057	-.012	1.000	.148	-.033	**-.368**	.167	.018
IL-6 (pg/ml)	-.141	-.019	.117	-.144	-.174	-.023	-.036	-.018	-.025	.047	.090	.148	1.000	-.027	-.123	.066	-.178
IL-1β (pg/ml)	-.146	-.138	.045	-.164	**.389**	.162	-.019	.008	.201	-.076	-.011	-.033	-.027	1.000	-.255*	-.014	.095
IL-10 (pg/ml)	.096	.212	-.244	.171	-.004	-.185	-.068	.182	-.220	-.129	-.011	**-.368**	-.123	-.255*	1.000	-.147	-.065
TNF-α (pg/ml)	-.037	-.222	.024	-.079	.023	.092	.118	-.192	.199	.225	.115	.167	.066	-.014	-.147	1.000	.163
TGF-β (pg/ml)	.322*	-.288*	.052	-.105	-.054	-.020	.305*	-.119	.151	.083	.040	.018	-.178	.095	-.065	.163	1.000

MDA, malondialdehyde; SOD, superoxide dismutase; 8-OHdG, 8-hydroxy-2′-deoxyguanosine; IL, interleukin; TNF-α, tumor necrosis factor-alpha; TGF-β, transforming growth factor-beta. Bold values indicate statistically significant correlations (p < 0.05, two-tailed). Correlation coefficients were calculated using Spearman’s rank correlation analysis.

## Discussion

This study evaluated the relationship between serum trace-element profiles, oxidative-stress biomarkers, and inflammatory cytokines in women with endometriosis. The findings demonstrate coordinated alterations in trace-element status, redox homeostasis, and inflammatory signalling. Women with endometriosis showed higher levels of arsenic, lead, mercury, copper, and iron and lower levels of zinc and selenium compared with healthy controls. These alterations were accompanied by increased MDA, SOD, and 8-OHdG and reduced glutathione, indicating substantial disruption of redox balance. Together, these findings provide further insight into the redox and inflammatory alterations associated with endometriosis.

Previous studies have reported altered trace-element profiles in women with endometriosis. Pascolo et al. observed accumulation of iron and potentially toxic environmental elements in ovarian endometriotic lesions, supporting a possible contribution of metal-associated oxidative injury to disease pathology (7). Other studies have reported alterations in zinc, copper, and selenium in affected women (5, 14). These observations are broadly consistent with our findings and suggest that both essential and potentially toxic trace elements may be altered in the context of endometriosis. Differences between studies may reflect variations in dietary intake, environmental exposure, occupational exposure, geographical characteristics, and analytical methods. Although smoking and alcohol abuse were excluded through the eligibility criteria, detailed dietary, occupational, and environmental exposure histories were not comprehensively quantified and may have influenced the observed serum trace-element concentrations (15).

Oxidative-stress profiling further demonstrated marked differences between women with endometriosis and controls (16). MDA and 8-OHdG were significantly increased, indicating greater lipid peroxidation and oxidative DNA damage, respectively, whereas glutathione was significantly reduced. SOD was also significantly elevated (17). The concurrent increase in SOD and reduction in glutathione may reflect differential responses of enzymatic and non-enzymatic antioxidant defenses under oxidative stress. These findings are consistent with previous reports describing oxidative imbalance in endometriosis and support the involvement of disrupted redox homeostasis in disease-associated cellular stress (18, 19).

The inflammatory profile showed significantly increased TNF-α, IL-1β, IL-6, and IL-10 levels, together with reduced IFN-γ, whereas TGF-β showed a non-significant increasing trend (20–22). The elevation of TNF-α, IL-1β, and IL-6 is consistent with the persistent inflammatory environment characteristic of endometriosis and may contribute to inflammatory signaling, lesion establishment, and tissue remodeling (23). The reduction in IFN-γ may reflect altered immune regulation, while increased IL-10 may represent a compensatory anti-inflammatory response (24). However, these interpretations should be considered cautiously because cytokine concentrations were measured at a single time point.

The correlation analysis identified several selective associations between trace elements and inflammatory or antioxidant biomarkers. Arsenic and zinc were positively correlated with TGF-β, while copper showed a moderate positive correlation with IL-1β. Iron showed a weak negative correlation with glutathione (25). These associations suggest that differences in trace-element status may be related to specific inflammatory and antioxidant responses in endometriosis. However, because this was a cross-sectional case-control study, the observed trace-element alterations cannot determine whether these abnormalities contribute to the development of endometriosis or arise as a consequence of the disease process. The observed associations may also be influenced by dietary intake, environmental exposure, disease characteristics, or other unmeasured factors (26).

The negative correlations involving lead require particularly cautious interpretation. Although lead concentrations were significantly higher in women with endometriosis, lead showed negative correlations with MDA and TGF-β (27). These associations should not be interpreted as evidence of a protective effect of lead. Rather, they may reflect complex biological regulation, differences in exposure patterns, or residual confounding. Longitudinal studies incorporating detailed environmental and nutritional exposure assessment will be important to clarify the significance of these relationships.

Additional associations were observed between oxidative and inflammatory biomarkers. 8-OHdG showed a significant negative correlation with IL-10, while IL-1β showed a weak negative correlation with IL-10 (28). These findings may indicate an association between oxidative DNA damage, inflammatory signalling, and anti-inflammatory regulation. Nevertheless, the strength of these correlations was modest, and the exploratory nature of the multiple pairwise comparisons require cautious interpretation (29). Previous studies have also reported increased 8-OHdG in women with endometriosis, supporting the relevance of oxidative DNA damage in the disease context (30).

The coexistence of trace-element imbalance, oxidative damage, altered antioxidant defenses, and inflammatory cytokine dysregulation highlights the interconnected molecular mechanisms underlying endometriosis and supports the role of integrated biomarker profiling in improving disease characterization (31, 32). hese findings provide hypothesis-generating evidence regarding the molecular interplay between trace-element homeostasis, oxidative stress, and inflammatory signaling in endometriosis. Future longitudinal and mechanistic studies are warranted to validate these associations and evaluate their clinical utility (33, 34).

## Limitations of the study

This study has several limitations. First, the sample size was relatively modest and derived from a single-center population, which may limit generalizability. Second, the endometriosis group was significantly younger than the control group (29.13 ± 5.44 vs. 32.40 ± 8.44 years; p = 0.013), and age may influence trace-element homeostasis, oxidative-stress responses, and inflammatory cytokine profiles. Consequently, residual confounding by age cannot be excluded, and future studies should incorporate age-matched cohorts or multivariable adjustment to better isolate disease-specific effects. Third, all women with endometriosis in this cohort presented with infertility, whereas the control group consisted of fertile women. Although this approach reduced the likelihood of including undiagnosed endometriosis among controls, it may introduce selection bias because some observed biomarker differences may relate to infertility status rather than endometriosis alone. Fourth, detailed dietary intake, environmental exposure, and occupational exposure histories were not comprehensively assessed, although smoking and alcohol abuse were excluded through the eligibility criteria. These unmeasured exposure factors may have influenced serum trace-element concentrations. Fifth, the cross-sectional case-control design precludes determination of temporal or causal relationships between trace-element imbalance, oxidative stress, inflammatory dysregulation, and endometriosis. Finally, no formal correction for multiple pairwise comparisons was applied because the correlation analysis was exploratory; therefore, these associations should be interpreted cautiously and validated in larger independent cohorts.

## Conclusion

Women with endometriosis demonstrated coordinated alterations in trace-element status, redox homeostasis, and inflammatory signaling. Elevated As, Pb, Hg, Cu, and Fe and reduced Zn and Se were accompanied by increased MDA, SOD, and 8-OHdG and reduced glutathione levels, together with significant alterations in inflammatory cytokines. Selective associations among trace elements, oxidative-stress biomarkers, and inflammatory cytokines further support an interconnected redox-inflammatory phenotype in endometriosis. Collectively, our findings demonstrate that disrupted trace-element homeostasis is closely associated with oxidative stress and inflammatory dysregulation in women with endometriosis. The integrated biomarker profile observed in this study supports the concept that interactions between toxic and essential trace elements, oxidative stress markers, and cytokines contribute to the systemic molecular landscape of endometriosis. These findings provide hypothesis-generating evidence for future mechanistic and longitudinal studies and highlight the potential value of integrated biomarker profiling for improving disease characterization and identifying potential therapeutic targets.

## Data Availability

The raw data supporting the conclusions of this article will be made available by the authors, without undue reservation.
